# 

**Published:** 1865-01

**Authors:** 


### INDEX TO VOLUME XIX.

Acknowledgment, 37
Annual Meeting of the Michigan Dental Association, 65
Address before the Michigan Dental Association, 70
Annealing Lamp, 76
A Letter, 86
Alveolar Periostitus, 167
Anæsthetics, 171
Amalgam, 194
A case. Its treatment, 197
Address of the retiring President, Dr. A. A. Blount, 199
Action of Iodine, and Iodide of Potassium on the Nervous system, 243
Action of Alcohol, 243
Anæsthesia by chemically pure ether, 245
A powerful disinfectant, 247
American Dental Convention, 249
American Dental Association, 250
A New Society, 250
An Address, 264
Address of the retiring President of the Buffalo Dental Association, 270
A new application of Electricity, 283
Action of Creosote in the treatment of Sycosis, 284
A few words on Bantingism and Obesity, 293
Announcement, Medical College, 299
Announcement, Dental College, 299
An address of welcome to the American Dental Association at Chicago, 324
A call to organize a Society, 345
A New Dental College, 392
Acquirements for admission into the Dental Profession, 402
Arsenic, 478
Annual meeting of the Merrimac Valley Dental Association, 480
Atlantic & G. W. R. R. 484

Buffalo Dental Association, 12, 209, 221
Baron Liebig's Soup for Children, 240
Bleaching Sponge, 245
Bleaching Teeth, 289

Connecticut State Dental Association, 23
Case of Necrosis of the Bones of the Head, 27
Central New-York Dental Association, 163
Correspondence, 232
Central Ohio Dental Society, 347, 446
Carroll's Literary Register, 300

Dental Therapeutics, 1
Death from Chloroform, 35, 244
Dental Circular & Examiner, 38
Dental Specialties, 74
Discussions at the Twenty-First Annual Meeting of the Mississippi Valley Dental Association, 100
Dental Fees, 187
Death of Dr. J. B. Smith, 251
Deciduous Teeth.—Their Importance; Their Preservation, 342

Extracting Teeth, 48
Effect of Tobacco on the Mental Powers, 244
Effects of Sugar on the Teeth, 245
Editorial, 249, 298, 346, 392, 439, 484
Essay read before the Iowa State Dental Association, 398
Extraction of Teeth without the Lancet, 441

Fees for Dental Pupilage, 191
Facial Neuralgia, 284

Going Abroad, 164

Hygiene R. R. Traveling, 247

Influence of Periostum on the Reproduction of Bone, 32
Illusions from the administration of Chloroform during Menstration, 246
Irritation of the Fauces with pain in the Shoulders and Thorax, caused from periostial Inflamation of the Teeth, 281
Influence of the Viscera of the Human Body upon the Dental Organs, 395

Kerosene for Heat in Vulcanizing, etc., 11

Louisville Dental Association, 221
Letter from H. J. McKellops, Paris, 232
Lancing the Gums of Infants, 353
Lancing the Gums in Extraction, 355

Medals *vs.* Patents, 19
Meeting of the Iowa State Dental Society, 54
Mechanical Dentistry, 79
Morality and Religion, 94
Minutes of the Twenty-First Annual Meeting of the Mississippi Valley Association of Dental Surgeons, 156
Mississippi Valley Dental Society, 165
Massachusetts Dental Society, 218
Mad River Valley Dental Association, 220
Medical Specialties, 346
Medical Schools, 437
Medical Department of the Army, 437

Nerve Instrument, 37
New Dental Society, 165
Notice, 163, 248
New Teeth, 252
New Instruments and Appliances, 298
New-York State Dental Delegation, 297
Nitrous Oxide, 433
New Test for Arsenic, 483

Ohio State Dental Society, 485

On Teething of Infants, 439

On the use of Chloroform and Ether in Dental Operations, 332

On Dental Students, 255

On the Degeneracy of the teeth, 258

Ohio College of Dental Surgery, 299

Obituary of Dr. S. Dunham, 77

Old Age, 24

Physiological Effects of Tobacco, 15
Poisoned by Tobacco juice, 22
Pain in Dental Surgery, 39
Proceedings of the Ohio Dental College Association, 202
Proceedings of the Cincinnati Academy of Medicine, 222
Preservation of the remains of Extinct Species, 241
Phosphates in Medical Decoctions and Infusions, 246
Perfect Anæsthesia, 246
Pleasures and Perplexities of Dental Practice, 338
Professional Etiquette, 249
Proceedings of the Iowa State Dental Society, 406, 358
Personal, 484, 393
Phosphor — Necrosis, 436
Paralysis of Arm resulting from a carious tooth, 481
Pecuniary, 486

Researches on the Medical Properties and Applications of nitrous-oxide Protoxide of Nitrogen or Laughing Gas, 440
Resolutions of the Mad River Valley Dental Association, 438
Retiring Address of Dr. S. B. Palmer, Pres. of the Central New-York Dental Society, 278
Report of the Chicago Dental Society, 215
Resignation of the Faculty, 254
Reorganization, 254
Refining scraps — Gold, Platinum, etc., 21
Removal of the Inferior Maxilla, 28
Reorganization, 37

Sir. B. C. Brodie, 237
Sharp-Sticks, 404
Smoking, 481
Subjects for Discussion, 482, 486
Selections, 447, 433

Teaching, 447
The Food and the Teeth, 455
The Ohio College of Dental Surgery, 439
The Steam Guage for Vulcanizing, 393
The Iowa State Dental Society, 394
The American Dental Association, 346
The American Dental Convention, 348
Teeth, and the importance of their preservation, 267
The Miami Medical College, 298
Transactions of the Central States Dental Association, 211
Transactions of the American Dental Association, 249
The Ohio Dental College Association, 253
The Steam Guage in Vulcanizing, 90
Treatment of Irregularity, 166
The use of the File, 51
Tongue Compressor, 77
Twenty-First Anniversary of the Mississippi Valley Dental Association, 10
The efficacy of Phosphate of Lime, 14
Testing Vulcanizers, 22
The Meetings, 38

Western Dental Society, 386

Wounds on the Face, 391

